# Fístula arteriovenosa após termoablação com laser endovenoso 1470 nm: relato de caso

**DOI:** 10.1590/1677-5449.003516

**Published:** 2016

**Authors:** Walter Junior Boim de Araujo, Adriano Carvalho Guimarães, Ricardo Herkenhoff Moreira

**Affiliations:** 1 Universidade Federal do Paraná – UFPR, Departamento de Cirurgia, Curitiba, PR, Brasil.; 2 V&P Day Hospital, Santo Antônio da Platina, PR, Brasil.; 3 Hospital Nossa Senhora da Saúde, Santo Antônio da Platina, PR, Brasil.

**Keywords:** varizes, terapia a *laser*, ultrassonografia, fístula arteriovenosa

## Abstract

O tratamento tradicional da insuficiência da veia safena magna (VSM) inclui a ligadura alta na junção safeno-femoral combinada com a fleboextração. No entanto, a morbidade associada à insatisfação do paciente com esse tratamento tem conduzido ao desenvolvimento de técnicas alternativas, e a termoablação com *laser* endovenoso (EVLT) tornou-se uma alternativa minimamente invasiva à cirurgia. A formação de fístula arteriovenosa (FAV) durante o EVLT é extremamente rara. Neste estudo, relatamos um caso de identificação ecográfica de FAV entre um segmento da veia safena acessória lateral e a artéria femoral superficial. Optou-se inicialmente pela realização de duas tentativas de compressão com transdutor linear, sem sucesso, e alternativamente o procedimento cirúrgico foi realizado sem intercorrência e com resolução da FAV. Esse relato de caso evidencia a importância do seguimento de vigilância ecográfica após o EVLT tanto para o controle da efetividade do método como para o diagnóstico e tratamento precoce de suas complicações.

## INTRODUÇÃO

A insuficiência venosa crônica causada por varizes é uma condição médica comum com taxas de prevalência de até 28 a 35% em adultos^1^. O tratamento tradicional da insuficiência da veia safena magna (VSM) inclui a ligadura alta na junção safeno-femoral combinada com a fleboextração. No entanto, a morbidade associada à insatisfação do paciente com esse tratamento tem conduzido ao desenvolvimento de técnicas alternativas^2^.

O primeiro estudo sobre a termoablação com *laser* endovenoso (EVLT) foi publicado por Charles Boné em 1999, com uso de *laser* diodo 810 nm^3^. No entanto, somente em 2001, quando Min, Navarro e Boné publicaram o primeiro trabalho relevante sobre o *laser* endovenoso para tratamento da VSM, essa técnica chamou atenção da comunidade de flebologistas^4^. Muitos estudos foram publicados posteriormente e, desde então, o *laser* endovenoso, cujo objetivo é destruir irreversivelmente a veia com refluxo, tornou-se uma alternativa minimamente invasiva à cirurgia.

Após a maior frequência de utilização do método associado ao melhor entendimento da tecnologia e o aprimoramento de novas técnicas, verificou-se uma queda na taxa de complicações do *laser* endovenoso. A formação de fístula arteriovenosa (FAV) durante a termoablação endovenosa é extremamente rara.

Neste estudo, em uma análise retrospectiva de 567 safenas submetidas a EVLT 1470 nm em um período de quatro anos, relatamos um caso de identificação ecográfica de FAV entre um segmento da veia safena acessória lateral (VSAL) e a artéria femoral superficial (AFS).

## DESCRIÇÃO DO CASO

Paciente do sexo feminino, 55 anos de idade, obesa, com índice de massa corporal (IMC) 34 e ausência de outras comorbidades. Apresentou-se na primeira avaliação com quadro clínico de varizes nos membros inferiores com classificação *Clinical-Etiology-Anatomy-Physiopathology* (CEAP) C3 e história de duas cirurgias prévias de varizes realizadas em outra instituição, associado ao quadro de lipedema e linfedema grau II. Ao eco-Doppler colorido (EDC) foi constatada a presença de segmentos venosos no trajeto da VSM direita, insuficiência da VSAL esquerda, múltiplas perfurantes insuficientes e varizes bilateralmente.

A paciente foi submetida a raquianestesia e colocada em decúbito dorsal, e posteriormente foi realizada punção ecoguiada da VSAL e passagem de fibra nua (*bare fiber*) a até 3 cm da junção safeno-femoral. Nesse momento, foi feita a tumescência com soro fisiológico em temperatura ambiente e efetuada a EVLT 1470 nm e densidade de energia linear endovenosa de 70-80 J/cm com bom controle ecográfico imediato. Também foram realizadas a termoablação de perfurantes e a retirada das varizes tributárias.

A paciente recebeu esquema profilático com enoxaparina de 40 mg, iniciado após seis horas do término da cirurgia e mantido por 7 dias. Foi estimulada a deambulação a partir da recuperação anestésica, e a paciente recebeu alta após 10 horas do ato operatório.

**Figura 1:**
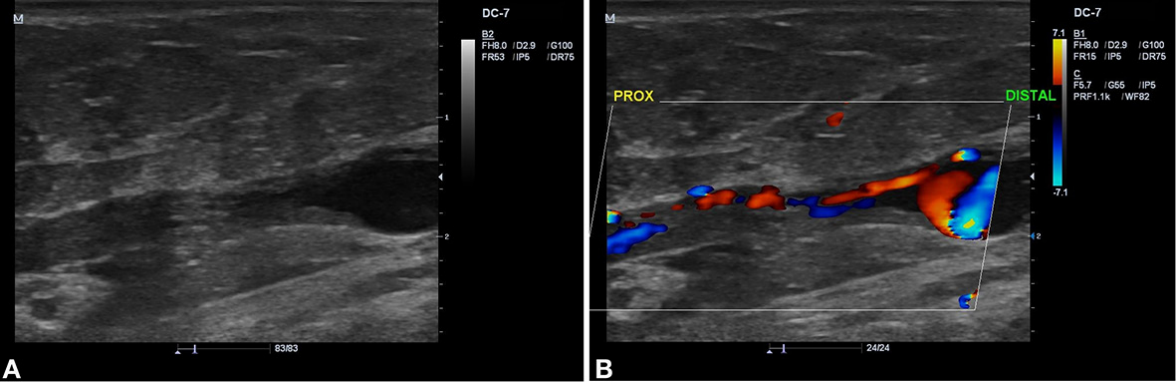
Imagem ecográfica evidenciando a recanalização do segmento médio-distal da veia safena acessória lateral (VSAL). (A) Modo B; (B) Modo color.

**Figura 2:**
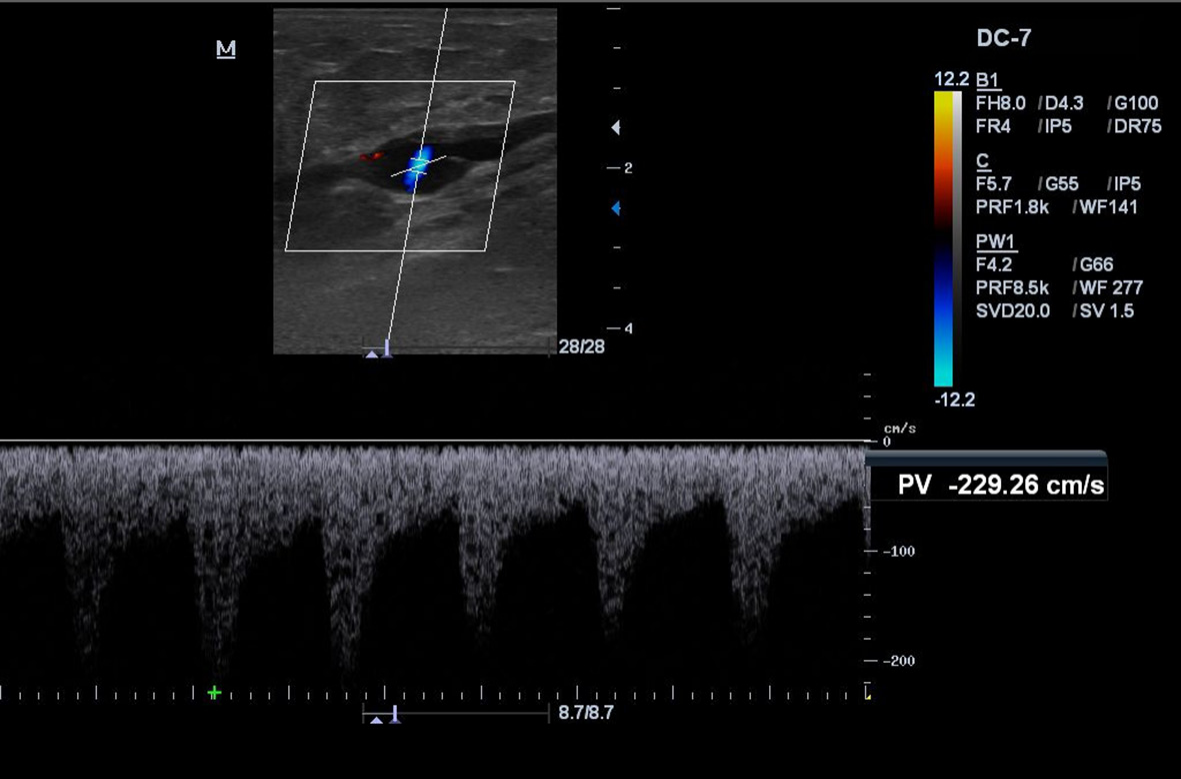
Imagem ecográfica do segmento médio-distal da veia safena acessória lateral (VSAL) evidenciando fluxo pulsátil de baixa resistência, com fluxo ascendente contínuo e sem interferência do ritmo respiratório, da manobra de Valsalva ou da compressão distal.

**Figura 3:**
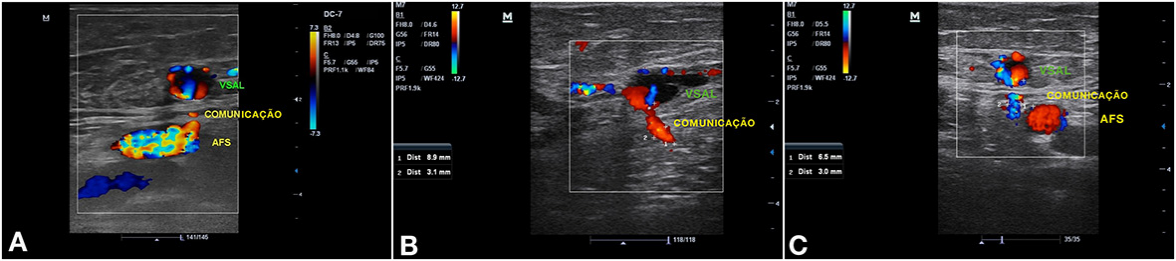
(A, B, C) Imagens ecográficas da fístula arteriovenosa (FAV) entre a veia safena acessória lateral (VSAL) e a artéria femoral superficial (AFS).

Evoluiu no primeiro retorno de acompanhamento ecográfico (7 dias) com oclusão da VSAL e das perfurantes tratadas, além da melhora do edema nos membros inferiores. No 45º dia, retornou para reavaliação clínica e realização do EDC de controle, quando se observou recanalização no segmento médio-distal da VSAL a partir de 8-10 cm da prega inguinal no sentido caudal (Figura 1). Também se identificou fluxo pulsátil de baixa resistência, com fluxo ascendente contínuo e sem interferência do ritmo respiratório, da manobra de Valsalva ou da compressão distal (Figura 2), compatível com FAV de alto débito entre a VSAL e a AFS (Figura 3). Nos segmentos abaixo e acima da FAV, a VSAL apresentava-se não compressível e com ausência de fluxo luminal.

Inicialmente, optou-se pela realização de compressão durante uma hora com transdutor linear, guiada por EDC. O mesmo procedimento foi repetido novamente após uma semana, sem desfecho favorável.

**Figura 4:**
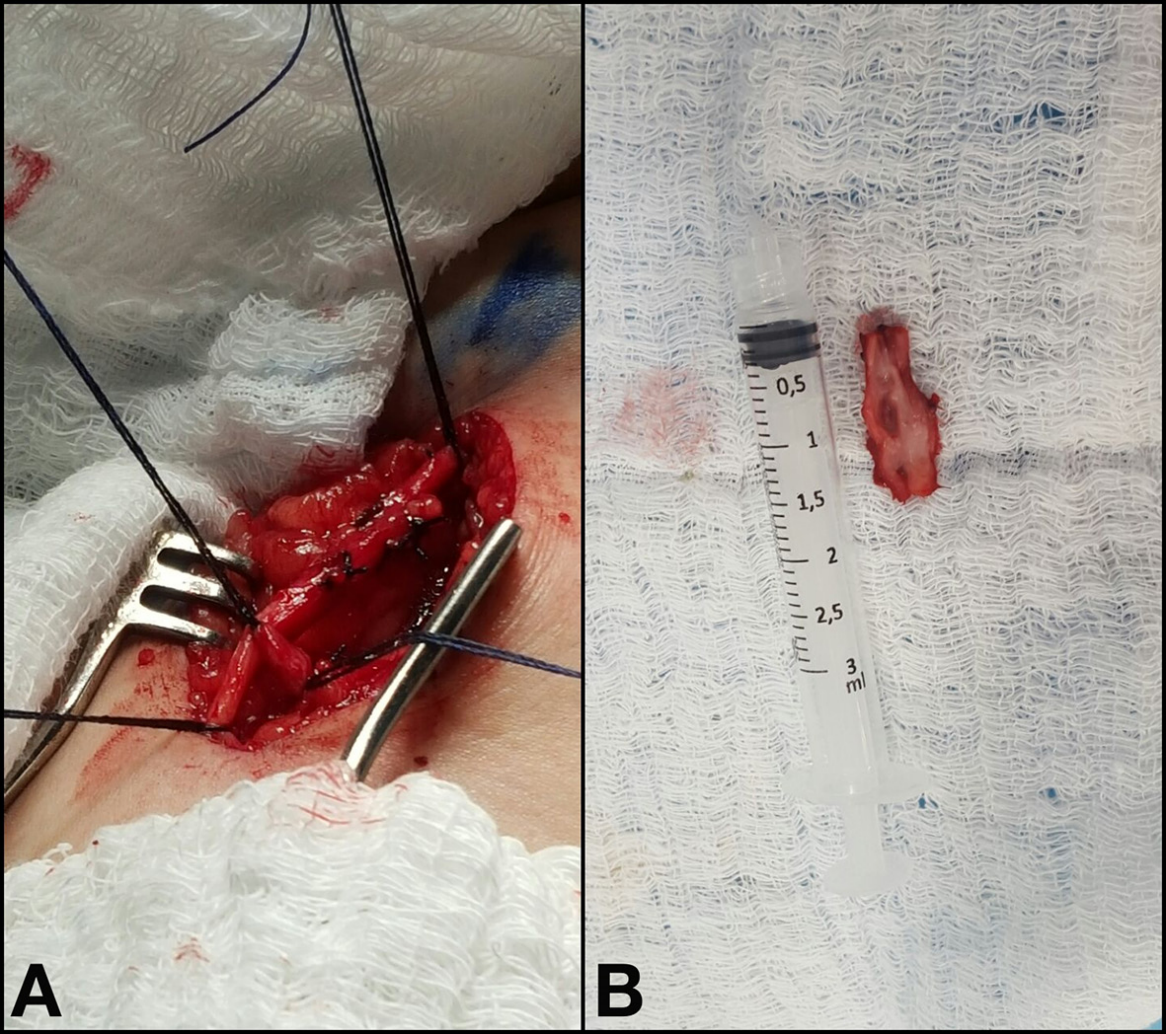
(A) Procedimento cirúrgico com abordagem proximal e distal do segmento da veia safena acessória lateral (VSAL) envolvido na fístula arteriovenosa (FAV), ligaduras das tributárias e identificação da FAV; (B) Segmento de VSAL envolvido na FAV submetido a exérese.

Depois disso, optou-se por abordagem cirúrgica da FAV com incisão guiada por EDC. Ao inventário cirúrgico observou-se veia de aspecto endurecido com fluxo pulsátil em pequeno segmento e múltiplas tributárias de fino calibre. Optou-se pela abordagem proximal e distal do segmento da VSAL envolvido na FAV, ligaduras das tributárias, identificação da FAV e posterior sutura do orifício com fio de prolene 4-0, além da exérese do segmento venoso residual (Figura 4).

**Figura 5:**
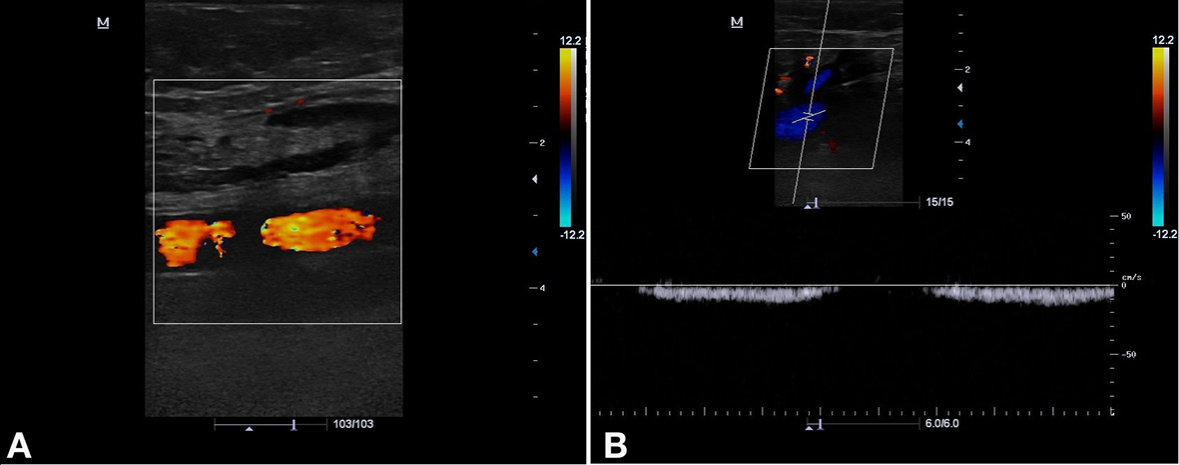
(A) Imagem ecográfica de controle evidenciando fluxo normal na artéria femoral superficial (AFS); (B) Imagem ecográfica de controle evidenciando fluxo normal na veia femoral comum.

O procedimento evoluiu sem intercorrências, e a paciente foi reavaliada no sétimo dia de pós-operatório, apresentando-se sem queixas e com boa evolução da ferida operatória. O EDC de controle evidenciou fluxo normal na AFS e na veia femoral comum (Figura 5).

## DISCUSSÃO

Bueno et al., em um estudo de série de casos, concluíram que a utilização do laser 1470 nm mostrou-se um bom método para tratamento das veias safenas^5^. Araujo et al.^6^, também realizando EVLT com o comprimento de onda 1470 nm, evidenciaram que, com a utilização de baixa densidade de energia, a incidência de complicações pode ser reduzida sem afetar significativamente os resultados clínicos.

A formação de FAV durante a termoablação endovenosa é extremamente rara. Theivacumar et al., analisando os dados de 1.500 doentes submetidos a termoablação endovenosa com laser 810 nm, descreveram três pacientes (< 0,2%) que desenvolveram FAV, sendo um após a termoablação da VSM e dois após a termoablação da veia safena parva. Os pacientes foram submetidos inicialmente a tratamento conservador, sendo que dois tiveram a resolução espontânea em 12 semanas e um persistiu com a fístula patente, porém com redução das velocidades de picos sistólicos durante 18 meses de acompanhamento^7^.

Rudarakanchana et al.^8^, em uma revisão realizada em 2011, relataram 11 casos de FAV descritos na literatura após a termoablação venosa, sendo a maioria deles após EVLT. Os sintomas dos pacientes após a formação da FAV variaram muito. Seis dos 11 pacientes não tiveram sintomas, e sua fístula foi encontrada na realização do EDC de rotina. Apenas três pacientes queixaram-se de edema na perna. Um paciente, que desenvolveu uma FAV na veia femoral comum de alto débito, apresentou-se com insuficiência cardíaca descompensada. A maioria das FAVs foram detectadas nos primeiros 30 dias de tratamento.

Hashimoto et al.^9^ descreveram um caso de uma paciente feminina de 64 anos de idade com história prévia de EVLT que se apresentou com quadro de insuficiência cardíaca de alto débito cardíaco causado por uma FAV femoral direita. Foi submetida a cirurgia aberta com resolução completa da FAV entre a AFS e a veia femoral superficial direita.

Embora a causa da formação da FAV durante a ablação por via endovenosa permaneça desconhecida, existem duas etiologias prováveis que geralmente são consideradas: lesões venosas e arteriais concomitantes durante a realização da tumescência; ou a transmissão de energia térmica através da parede da veia para a artéria vizinha, levando à degradação da parede vascular tardiamente e formação de uma FAV. Certas considerações técnicas, se não seguidas corretamente, podem aumentar o risco. É importante utilizar um alto volume de líquido para tumescência nas áreas mais críticas para afastar quaisquer ramos arteriais, bem como para dissipar o calor durante a ablação. A punção e a infiltração com líquido tumescente devem ser guiadas por ultrassom^8^.

A literatura não parece apoiar o tratamento cirúrgico, e sim a observação clínica da maioria das FAVs associadas a EVLT. Esses pacientes podem ser seguidos através de exames clínicos e EDCs seriados. Exames de imagem mais invasivos e o tratamento cirúrgico ou endovascular devem ser reservados para pacientes que se tornam sintomáticos^8^.

No caso descrito – uma paciente assintomática com achado de FAV no acompanhamento ecográfico de rotina –, acredita-se que a causa da formação da FAV provavelmente está associada a lesão iatrogênica durante a realização da tumescência perivenosa. Optou-se inicialmente pela realização de duas tentativas de compressão com transdutor linear no intervalo de uma semana, mas sem sucesso, provavelmente pelo fato de se tratar de FAV de alto débito. Alternativamente, o procedimento cirúrgico foi realizado sem intercorrência e com resolução da FAV.

## CONCLUSÃO

Com a disseminação das técnicas de termoablação endovenosa e o consequente aumento do número de procedimentos realizados, têm sido descritas complicações que, embora raras, até então não eram descritas com a utilização do tratamento clássico da fleboextração cirúrgica. O relato deste caso evidencia a importância do seguimento de vigilância ecográfica após o tratamento de termoablação endovenosa tanto para o controle da efetividade do método como para o diagnóstico e tratamento precoce de suas complicações.
