# Supplementary material for: Fístula arteriovenosa após termoablação com laser endovenoso 1470 nm: relato de caso
Source: J Vasc Bras. 2016 Jul-Sep;15(3):254–8. [Article in Portuguese] doi: 10.1590/1677-5449.003516 (PMC5829765; doi:10.1590/1677-5449.003516)
Supplement: Translation

# Translation: Arteriovenous fistula after endovenous laser ablation with 1470 nm laser: case report

**Authors:** Walter Junior Boim de Araujo, Adriano Carvalho Guimarães, Ricardo Herkenhoff Moreira

**Affiliations:** 1 Universidade Federal do Paraná – UFPR, Departamento de Cirurgia, Curitiba, PR, Brazil.; 2 V&P Day Hospital, Santo Antônio da Platina, PR, Brazil.; 3 Hospital Nossa Senhora da Saúde, Santo Antônio da Platina, PR, Brazil.

**Keywords:** varicose veins, laser therapy, ultrasonography, arteriovenous fistula

## Abstract

Traditional treatment for great saphenous vein (GSV) insufficiency includes high ligation of the saphenofemoral junction and subsequent stripping of the GSV. However, the considerable morbidity and patient dissatisfaction associated with surgical treatment led to development of alternative techniques and intravenous laser treatment (EVLT) has emerged as a minimally invasive alternative to surgery. Formation of arteriovenous fistulas (AVF) during EVLT is extremely rare. In this study we report a case of AVF between a segment of the lateral accessory saphenous vein and the superficial femoral artery that was identified by ultrasound. Initially, two attempts were made at compression with a linear-array transducer without success, then alternatively surgery was performed without complications, leading to resolution of the AVF. This case report highlights the importance of ultrasound follow-up after EVLT, both for monitoring the effectiveness of the method and for diagnosis and early treatment of its complications.

## INTRODUCTION

Chronic venous insufficiency caused by varicose veins is a common medical condition with prevalence rates as high as 28 to 35% among adults.^1^ The traditional treatment for great saphenous vein (GSV) insufficiency includes high ligation at the saphenopopliteal junction combined with stripping. However, morbidity and patient dissatisfaction associated with the treatment have led to development of alternative techniques.^2^

The first study describing endovenous laser thermal ablation (EVLT), using an 810 nm diode laser, was published by Charles Boné in 1999.^3^ However, it was only after Min, Navarro and Boné published the first relevant study about endovenous laser for treatment of GSV in 2001 that the technique came to the attention of the phlebologist community.^4^ Many more studies were published afterwards and the use of endovenous lasers to irreversibly destroy veins with reflux became a minimally invasive alternative to surgery.

After the frequency with which the method was used increased, in combination with improving understanding of the technology and the development of newer and better techniques, the rate of complications associated with endovenous laser treatment began to fall. Formation of an arteriovenous fistula (AVF) during endovenous thermal ablation is extremely rare.

This study describes a case of an AVF detected sonographically between a segment of the lateral accessory saphenous vein (LASV) and the superficial femoral artery (SFA) that was identified during a retrospective analysis of 567 saphenous veins treated with 1470 nm EVLT over a period of 4 years.

## CASE DESCRIPTION

The patient was a 55-year-old female with obesity and a body mass index (BMI) of 34, free from other comorbidities. At the initial assessment she presented with varicose veins of the lower limbs with a clinical classification of C3 according to the Clinical-Etiology-Anatomy-Physiopathology (CEAP) C3 framework and a medical history including two prior surgeries for varicose veins conducted at other institutions and class II lymphedema and lipedema. Color Doppler ultrasonography (CDUS) showed presence of venous segments along the path of the right GSV, insufficiency of the left LASV, multiple insufficient perforating veins and bilateral varicose veins.

Spinal anesthesia was administered and the patient was placed in decubitus dorsal and then ultrasound-guided puncture of the LASV was performed followed by insertion of a bare fiber which was advanced up to a point 3 cm from the saphenopopliteal junction. At this point, tumescence was achieved with saline at room temperature and then 1470 nm EVLT was performed at a linear endovenous energy density of 70-80 J/cm, with good results according to control ultrasound conducted immediately afterwards. Thermal ablation of perforating veins was also performed and tributary varicose veins were stripped.

The patient was prescribed a prophylactic course of 40 mg enoxaparin, started six hours after the end of surgery and continued for 7 days. She was encouraged to walk as soon as she had recovered from the anesthetic and was discharged 10 hours after the operation.

At a 7-day follow-up appointment, ultrasound showed occlusion of the LASV and of the perforating veins that had been treated and lower limb edema had improved. However, 45 days after surgery she returned for clinical reassessment and CDUS showed recanalization of the mid-distal segment of the LASV, starting 8-10 cm distal of the inguinal fold (Figure 1). Additionally, the examination detected low-resistance pulsating, continuous, and ascending flow that did not suffer interference from respiratory rhythm and did not respond to the Valsalva maneuver or to distal compression (Figure 2), compatible with a high throughput AVF between the LASV and the SFA (Figure 3). Segments of the LASV above and below the AVF were non-compressible and did not exhibit flow through the lumen.

**Figure 1:** Sonographic images showing recanalization of a mid-distal segment of the lateral accessory saphenous vein (LASV). (A) Mode B; (B) Color mode.

**Figure 2:** Sonographic image of the mid-distal segment of the lateral accessory saphenous vein (LASV) showing low resistance pulsating flow, with continuous ascending flow and no interference from respiratory rhythm or response to the Valsalva maneuver or distal compression.

**Figure 3:** (A, B, C) Sonographic images of the arteriovenous fistula (AVF) between the lateral accessory saphenous vein (LASV) and the superficial femoral artery (SFA).

Initially, compression was applied for 1 hour with the linear transducer guided by CDUS. The same procedure was repeated 1 week later, but was unsuccessful.

After this, the decision was taken to treat the AVF surgically via a CDUS-guided incision. Surgical examination revealed a vein with a hardened appearance and pulsating flow through a small segment, with multiple small-caliber tributaries. The decision was taken to approach the LASV segment involved in the AVF both proximally and distally, ligate the tributaries and, after identification of the AVF, suture its orifice with 4-0 prolene thread and excise the remnant venous segment (Figure 4).

**Figure 4:** (A) Surgical procedure with proximal and distal approach to the segment of the lateral accessory saphenous vein (LASV) involved in the arteriovenous fistula (AVF), with ligation of tributaries and identification of the AVF; (B) Excised segment of the LASV that was involved in the AVF.

This procedure was performed without any intercurrent conditions and the patient was reassessed 7 days later, when it was found that she was free from complaints and the surgical wound was healing well. The control CDUS showed normal flow in the SFA and in the common femoral vein (Figure 5).

**Figure 5:** (A) Control sonographic image showing normal flow in the superficial femoral artery (SFA); (B) Control sonographic image showing normal flow in the common femoral vein.

## DISCUSSION

Bueno et al. published a study describing a case series in which they concluded that ablation with a 1470 nm laser had proven to be a good method for treatment of saphenous veins.^5^ Araujo et al.^6^ also studied EVLT using the 1470 nm wavelength, showing that by employing a low energy density the incidence of complications could be reduced without significantly impacting the clinical results.

Formation of an AVF during endovenous thermal ablation is an extremely rare occurrence. For example, Theivacumar et al. analyzed data from 1,500 patients who underwent endovenous laser thermal ablation with a wavelength of 810 nm and only reported three patients (< 0.2%) who developed an AVF, one after thermal ablation of the GSV and two after thermal ablation of the small saphenous vein. These patients were initially managed conservatively and two of them enjoyed spontaneous resolution in 12 weeks, while the third’s fistula remained patent, but its peak systolic velocity reduced over 18-months of follow-up.^7^

In a review published in 2011, Rudarakanchana et al.^8^ reported 11 cases described in the literature of AVF after venous thermal ablation, the majority after EVLT. The symptoms displayed by patients after formation of the AVF varied greatly. Six of the 11 patients did not have symptoms and their fistulas were detected during routine CDUS. Just three patients complained of edema of the leg. One patient, who developed a high throughput AVF in the common femoral vein presented with decompensated heart failure. The majority of these AVFs were detected within 30 days of treatment.

Hashimoto et al.^9^ described the case of a 64-year-old female patient with a prior history of EVLT treatment who presented with high-output heart failure caused by a right femoral AVF. She was treated with open surgery that fully resolved the AVF between the SFA and the right superficial femoral vein.

While the causes of formation of an AVF during endovenous ablation are unknown, there are two probable etiologies that are generally taken into consideration: concurrent venous and arterial injuries during tumescence; and transmission of thermal energy through the wall of the vein into a neighboring artery, leading to later degradation of the vascular wall and formation of an AVF. Failure to observe certain technical precautions correctly can increase the risk. It is important to employ a large volume of liquid to provoke tumescence in the most critical areas to displace any arterial branches and to dissipate heat during ablation. Both puncture and infiltration of the tumescence liquid should be guided with ultrasound.^8^

For the majority of EVLT-related AVFs, the literature does not appear to support surgical treatment. Clinical observation is preferred instead. These patients can be monitored with serial CDUS and clinical examinations. More invasive imaging exams and surgical or endovascular treatment should be reserved for patients who become symptomatic.^8^

In the case described here – an asymptomatic patient whose AVF was found during a routine sonographic follow-up examination – it is believed that the cause of AVF formation was probably linked to a iatrogenic injury during the perivenous tumescence procedure. Initially, two attempts were made at compression with a linear transducer, separated by a 1-week interval, but they were unsuccessful, probably because of the high throughput of the AVF. As an alternative, a surgical procedure was performed with no complications and with successful resolution of the AVF.

## CONCLUSIONS

As techniques for endovenous thermal ablation have become more widespread, complications have been described that, although rare, had not been observed after classic surgical stripping treatment. This case report demonstrates the importance of follow-up with sonographic surveillance after treatment with endovenous thermal ablation, both to monitor the effectiveness of the method and to diagnose and treat its complications early.
